# Implications of Antioxidant Systems in Inflammatory Bowel Disease

**DOI:** 10.1155/2018/1290179

**Published:** 2018-05-09

**Authors:** Guiping Guan, Shile Lan

**Affiliations:** College of Bioscience and Biotechnology and College of Animal Science and Technology, Hunan Agricultural University, Changsha, Hunan 410128, China

## Abstract

The global incidence of inflammatory bowel disease (IBD), a group of chronic gastrointestinal disorders, has been rising. The preponderance of evidence demonstrates that oxidative stress (OS) performs a critical function in the onset of IBD and the manner of its development. The purpose of this review is to outline the generation of reactive oxygen species and antioxidant defense mechanisms in the gastrointestinal tract and the role played by OS in marking the onset and development of IBD. Furthermore, the review demonstrates the various ways through which OS is related to genetic susceptibility and the mucosal immune response. The experimental results suggest that certain therapeutic regimens for IBD could have a favorable impact by scavenging free radicals, reducing cytokine and prooxidative enzyme concentrations, and improving the antioxidative capabilities of cells. However, antioxidative activity characterized by a high level of specificity may be fundamental for the development of clinical therapies and for relapsing IBD patients. Therefore, additional research is required to clarify the ways through which OS is related to the pathogenesis and progression of IBD.

## 1. Introduction

The group of chronic disorders of the gastrointestinal (GI) tract known as inflammatory bowel disease (IBD), including its main types, Crohn's disease (CD) and ulcerative colitis (UC), is marked by sequential stages of symptom and sign disappearance (remission) followed by clinical relapse. Although the adverse effects of CD are not associated with a specific part of the GI tract, UC is characterized by its localization to the rectum and colon. Despite the unclear etiologic underpinning of IBD, the pathogenesis and progression of the inflammatory cascade in patients with CD and/or UC are often attributed to genetic, microbiome, and environmental factors [1].

Physiological oxygen metabolism gives rise to reactive oxygen/nitrogen species (ROS/RNS), which pose a threat to existing cells within the body. ROS and RNS function as signaling molecules in mitogenic responses or as safeguards against pathogens when observed at low-to-moderate concentrations, but oxidative stress (OS) and nitrosative stress (NS) arise when ROS scavenge in an inefficient manner or RNS are produced in excess. High concentrations of the two molecules can change the body's inflammatory response and bring about changes in lipids and proteins, paired with programmed cell death (apoptosis), cancerogenic cell transformation, and DNA lesions [2]. ROS are primarily produced by mitochondria, which possess dedicated ROS scavenging mechanisms (which aid in cell survival), in physiological and pathological states [3]. However, the literature indicates that mitochondria produce ROS at a level that is disproportionate to their scavenging capability [4].

Many researchers consider that OS, which is an immune-regulatory factor, is a principal agent in the pathophysiology of IBD. Studies have shown that prolonged inflammation of the intestines is linked to excessive ROS/RNS production, which, as previously noted, results in OS and NS, respectively. Both OS and NS have been found to be causative agents of various adverse health conditions, including IBD [5]. The literature also indicates that IBD is linked to disequilibrium between ROS and antioxidant activity, with impaired antioxidant activity or heightened ROS production, giving rise to OS [6].

Over the past 20 years, the pathological mechanisms of IBD have been increasingly documented in the medical literature. Nevertheless, owing to the adverse and unfavorable secondary effects that patients of modern IBD treatments regularly suffer, our understanding of the role played by OS in IBD must be improved and therapeutic interventions should thereby be enhanced [7]. Therefore, the purpose of this review article is to provide a summary of the most recent data pertaining to the role of OS as a causative agent in UC and CD.

## 2. Free Radicals in the Intestine

### 2.1. Reactive Oxygen Species

Various ROS arise as a consequence of the standard metabolic processes of biological systems, including superoxide radicals (O_2_^•−^), hydroxyl radicals (^•^OH), hydrogen peroxide (H_2_O_2_), and singlet oxygen (^1^O_2_) [3]. A range of physiological processes are dependent on minimal ROS levels, including protein phosphorylation, cell immunity, apoptosis, cell differentiation, and transcription factor activation [8]. Furthermore, ROS at low-to-moderate concentrations serve as secondary signals for the regulation of cardiac and vascular cell functioning [8]. The superoxide anion (O_2_^•−^), which results from the addition of one electron to molecular oxygen, is the free radical with the highest prevalence in human tissues. Its primary source in cells is complexes I and III of the mitochondrial electron transport chain, which is responsible for conversion of approximately 1% to 3% of total oxygen to O_2_^•−^ [9]. The enzymatic reaction catalyzed by xanthine oxidase (XO) and membrane enzyme complexes (NADPH oxidases [NOX]) represents an additional source of O_2_^•−^. In an environment characterized by stress, the concentrations of O_2_^•−^ increase to a point at which deleterious hydroxy radicals (OH^•^) are produced excessively by the Haber-Weiss reaction. In the GI tract, OH^•^ is responsible for inactivating a fundamental mitochondrial enzyme, pyruvate dehydrogenase, which results in the depolymerization of GI mucin [10], which in turn facilitates mitochondrial RNA and DNA damage. In addition to this, perhydroxyl (HOO^•^), a protonated form of O_2_^•−^ responsible for causing fatty acid peroxidation [11], is responsible for disrupting the degree to which biomembranes are fluid and permeable, thereby changing lipoproteins into proinflammatory forms and forming products that are potentially toxic [12].

Various intracellular organelles, including the endoplasmic reticulum, mitochondria, nucleus, peroxisomes, and the cytosol and extracellular matrix, are responsible for producing the majority of endogenous ROS [13]. The mitochondrial electron transport chain also plays a prominent role in the production of ROS [13]. In addition to mitochondria, plasma membrane NADPH oxidases and peroxisomes contribute to the number of cellular free radicals, giving rise to H_2_O_2_ as a result of oxygen consumption. Within the GI tract, O_2_^•−^ is primarily produced by XO, and in the context of pathological states, circulating XO undergoes binding to vascular endothelial cells, giving rise to site-specific oxidative injury of intestinal tissue [14]. It is noteworthy that the basal level of ROS in enterocytes is variable and that the ROS concentration is lower in the small intestine than in the colon [14]. This variability can affect the levels of oxidized protein and lipids, as well as DNA damage, thereby affecting the degree to which the GI tract is susceptible to GI diseases at various sites.

### 2.2. Reactive Nitrogen Species

RNS are a group of free radicals that are produced by nitric oxide synthases (NOS), which are expressed within the intestinal submucosa and mucosal regions. NOS are responsible for the metabolization of arginine to citrulline, which leads to the formation of nitric oxide radical (NO^•^) via a five-electron oxidative reaction [15]. Over the course of the progression of inflammatory diseases, NO steady-state levels within the bowel mucosa can be seriously affected by alterations in the synthesis and disposition of RNS. Improved phagocytic cell recruitment, followed by an increase in the concentration of inducible NOS (iNOS) (both of which take place in IBD-affected cellular mucosa), occurs alongside changes in the local endothelium, which leads to expression of iNOS and endothelial NOS (eNOS). The half-life of NO^•^ is quite long, but because of its quick diffusion into the bloodstream and subsequent inactivation owing to hemoglobin, its reaction time is slow [16]. Furthermore, NO^•^ protects epithelial cells against the toxicity that arises from H_2_O_2_ and impairs leukocyte adhesion to endothelial cells [17]. Although eNOS gives rise to NO^•^ via pulsation, iNOS generates it in a continuous manner. The literature shows that, in UC, iNOS/cyclooxygenase-2 (COX-2)/5-lipoxygenase (5-LOX) loop activation and increased amounts of their end products (i.e., NO, prostaglandin E_2_ [PGE_2_], and leukotriene B_4_ [LTB_4_]) damage the mucous membrane of the large intestine due to overproduction of free radicals and inhibition of the antioxidative system [18]. It is important to note that the inducible and constitutive variants of NO synthases that appear in standard intestinal microvasculature are significantly reduced in the context of IBD. Furthermore, the total quantity of NO in the inflamed gut mucosa over the long term was shown to be seriously high, as a result of tissue invasion by inflammatory cells [19], and the local concentration of iNOS is heightened in response [19]. In addition, iNOS-derived NO undergoes a reaction with tyrosine, thereby producing nitrotyrosine. Researchers have found that tissue from UC patients (but not those with collagenous colitis) is characterized by intense epithelial staining for nitrotyrosine, which is linked to the infiltration of neutrophils in the epithelium [20].

### 2.3. Targeting Oxidative Stress in IBD

IBD is typically diagnosed in pediatric and young adult patients [21]. UC inflammation is usually restricted to the colon's mucosa and submucosa and starts in the rectum before spreading to nearby areas (continuously and often involving the periappendiceal region) [22]. Proinflammatory cytokines, along with ROS and NOS, have long been implicated in the pathogenesis and development of UC [23]. In another study, the researchers observed notable neutrophil infiltration, paired with an increasing myeloperoxidase level in patients with UC [24]. Furthermore, iNOS-derived NO has been found to stimulate TNF-*α* production in the colon, which then causes neutrophil infiltration (e.g., via the promotion of intercellular adhesion molecule (ICAM) and P-selectin production, thus causing colonic tissue damage) [25]. Neutrophil recruitment and activation of important transcriptional pathways, including nuclear factor-kappa B (NF-*κ*B) and AP-1, also heighten the inflammatory response and tissue damage [24]. In addition, NF-*κ*B can facilitate the modulation of the permeability of the intestinal layer and is linked to intestinal epithelial cell homeostasis [26]. It is also notable that NF-*κ*B signaling can heighten the chronic intestinal inflammation that characterizes the mucosa in patients with UC [27]. Intestinal epithelial cells, which recognize microbial-activated toll-like receptors and TNF-*α*, facilitate the stimulation of NF-*κ*B signaling events, and it should be noted that these are responsive to OS [28, 29].

The hallmarks of CD include comparatively low levels of naïve T cells, higher levels of memory T cells, and elevated expression of major histocompatibility complex (MHC) class II molecules [30]. All areas within the GI tract can be affected by CD, but the terminal ileum or perianal region is usually affected [31]. In the preliminary phases of the condition, patchy necrosis of the surface epithelium can be detected, and this is paired with the detection of focal accumulations of leukocytes proximate to crypts and large numbers of intraepithelial macrophages and granulocytes. Stimulated inflammatory cells give rise to ROS and RNS, but the underlying mechanisms associated with the production of free radicals and their origins in CD patients are not straightforward. In recent years, the literature has indicated that immune peripheral cells in patients with active CD are characterized by greater superoxide dismutase (SOD) activity and H_2_O_2_ generation, paired with higher lipid peroxidation, impaired mitochondrial function, and impaired catalase (CAT) activity [32]. It is particularly noteworthy that these modifications, aside from CAT activity, are subject to reversal over the course of disease remission, which indicates the crucial role of mitochondria and OS in the progression of CD [32].

In addition to IL-4, cytokines such as TNF-*α*, IL-1*β*, IL-6, and IL-8 play an important role in CD [33]. Both ROS and RNS induce cytokine secretion. Notably, a recent study indicated that NOS mRNA was upregulated in peripheral blood mononuclear cells and the colonic mucosa in active CD patients, and it was hypothesized that NOS-derived NO^•^ and the plasma levels of some proinflammatory cytokines are positively correlated [34]. Furthermore, it is worth noting that NOX enzymes give rise to free radicals such as O_2_^•−^. In particular, in the presence of SOD3, O_2_^•−^ undergoes conversion to hydrogen peroxide, and/or it then results in a direct increase in the content of advanced glycation end products (AGEs). AGE paired with proinflammatory cytokines such as IL-6 or TNF-*α* activates the NF-kB signaling pathway. This results in the heightened expression of caspase 3, TNF-*α*, and IL-6 genes and activates MAPK, thereby resulting in the amelioration of AP-1 expression and increasing the level of iNOS. When considered together, the impairment of NF- *κ*B or p38 MAPK may lower cytokine production in the context of CD while influencing ROS/RNS production, particularly with respect to the condition's active phase [35].

### 2.4. Antioxidant Defenses in the GI Tract

Despite the manner in which unregulated OS in the GI tract exerts a detrimental effect on its continued and effective functioning, the antioxidant defenses of the human body can limit the negative outcomes that result from excessive ROS production. Various defense mechanisms are responsible for establishing appropriate ROS/RNS concentrations, and the endogenous antioxidant system primarily comprises intracellular enzymatic antioxidants, including SOD, glutathione peroxidase (GPx), and CAT; intracellular nonenzymatic antioxidant glutathione; and extracellular antioxidants (e.g., vitamins, minerals, and ceruloplasmin).

### 2.5. Intracellular Enzymatic Antioxidants

As reported in recent studies, enzymatic and nonenzymatic antioxidant cellular defense systems perform a critical function in safeguarding elements of the human body against ROS, and this occurs by controlling free radical and metabolite production [36, 37]. The key antioxidant enzymes oriented against superoxide radicals are SOD, CAT, and GPx, which perform in concert in the ROS metabolic pathway. It is important to recognize that lipid peroxidation can occur when the activity of one enzyme changes and when this is simultaneously unaccompanied by relevant modifications in its counterparts [36, 37].

Recent research indicates that the mucosa in patients with active CD or UC displays relatively low SOD levels [38]. It is noteworthy that SODs are enzymes that are oriented around a metal cofactor, which itself operates to convert O_2_^•−^ to O_2_ and H_2_O_2_ in a catalytic manner [4]. It is possible to categorize enzymes of this kind into the following four groups:

- Iron SOD (Fe-SOD), which is native to the chloroplasts of eukaryotic cells
- Manganese SOD (Mn-SOD), which is commonly identified in mitochondria (but can also be observed in peroxisomes)
- Copper-zinc SOD (Cu/Zn-SOD), which is the most abundant of all SODs, situated in chloroplasts, the cytosol, and extracellular space
- Nickel SOD (Ni-SOD), which has been isolated from several* Streptomyces* bacteria and cyanobacteria [39]

SOD activity stimulated by intrarectal acetic acid can be identified in patients with UC or CD, especially when compared to healthy controls, and it is possible to restore pronounced SOD levels to what is normally expected when patients are in remission [32]. Overall SOD activities are higher in IBD pathogenesis, because they constitute a way to safeguard intestinal tissues from oxidative damage, which results from inflammation and OS. The levels of SOD in IBD patients' peripheral blood are therefore currently being used as an OS biomarker.

The literature demonstrates that isoforms of GPx are upregulated in a transcriptional way by oxidative mechanisms and as a consequence of the OS response [40]. It is possible for GPx to catalyze glutathione into oxidized glutathione (glutathione disulfide [GSSG]), and it has the capability to reduce H_2_O_2_ to H_2_O or lipid hydroperoxides (ROOH) into stable alcohols. GPx, combined with glutathione reductase (GSSG-R), sustains reduced glutathione (GSH) levels and thus safeguards cells from peroxide damage. The following are the subspecies of GPx:

- GPx1: observed in the cytosol of most cells (inclusive of red blood cells)
- GPx2: observed in the cytosol but not found outside the GI tract
- GPx3: observed in plasma as a glycoprotein
- GPx4: observed in mitochondria, in which it engages with complex lipids (e.g., cholesterol and lipoproteins) which have been damaged by free radicals [41]

In a recent study, researchers examined a sample group of patients with UC in the active or remission stage and found that a noteworthy rise in GPx activity was associated with the inflamed mucosa. Additional research has verified that patients with UC and CD have higher plasma GPx levels than healthy controls, and it is particularly notable that this is true during both active and remission phases [42]. However, numerous contradictory findings have been released with respect to the antioxidant capabilities of SOD and GPx in IBD, depending on controls resulting from various approaches and conditions. In view of this, further research is necessary.

CAT, primarily situated in peroxisomes and responsible for catalyzing the reduction of H_2_O_2_ to H_2_O and O_2_ [43, 44], is mainly distributed in the liver, kidney, and erythrocytes. CAT can be categorized into the following types: (i) typical or monofunctional catalases (e.g., mammal-type catalases), (ii) bifunctional catalase-peroxidases, and (iii) pseudocatalases. Mammal-type catalases are characterized by their activity as tetramers, and they exhibit relatively reduced rates of peroxidase activity. Furthermore, their target molecules are restricted to microscopic organic substrates. Catalase-peroxidases are characterized by bifunctionality; they serve as both catalase and peroxidase. Furthermore, they can capitalize on numerous organic substances to serve as hydrogen donors. In stark contrast to monofunctional catalases, catalase-peroxidases are active as dimers or tetramers. Pseudocatalases, also referred to as non-heme manganese-containing catalases, are non-heme catalases that include three characterized and sequenced enzymes that hail from various bacterial species. It is possible for a collection of pathogens associated with GI tract conditions to cause modified CAT expression [45]. Reduced CAT activity is seen in patients with CD and in patients with colorectal cancer, gastric adenocarcinoma, and* H. pylori*-positive stomach ulcers [46, 47]. Furthermore, experimental studies of genetically altered mice have found that elevated CAT activity is linked to a decrease in the likelihood of developing colitis or colon cancer [48].

### 2.6. Intracellular Nonenzymatic Antioxidants

GSH is a notable intracellular nonenzymatic antioxidant. It is a water-soluble tripeptide that contains a cysteine-derived thiol group. The reduced form of GSH is prevalent in the cytoplasm, nucleus, and mitochondria. In each cell compartment, it has been found to serve as the main soluble antioxidant. The sustenance of GSH homeostasis in healthy tissues can occur as a consequence of de novo synthesis from cysteine, GSSG regeneration, and GSH uptake by way of sodium-dependent transport systems [49]. In addition, GSSG synthesis stems from the reduction of a pair of GSH particles when NADPH is present. GSH regeneration from GSSG takes place via the reaction facilitated by GSH reductase (GRd), or it is removed from the cell as a consequence of exportation into the extracellular space [50]. GSH, combined with glutathione peroxidase (GPx), glutathione reductase (GSr), and glutathione S-transferase (GST), establishes in the gut mucosa an antioxidant obstacle. It is notable that GSH is regularly used as a biomarker not only for inflammation but also for OS. It has been reported that a reduced GSH level is associated with dextran sulfate sodium- (DSS-) induced colitis and that antioxidant therapies can be applied to reestablish health [51].

Melatonin is an endogenous hormone that is primarily synthesized in and secreted by the pineal gland [52]. In the 1960s, melatonin was isolated and chemically identified as N-acetyl-5-methoxytryptamine [52]. The literature indicates that melatonin treatment limits the severity of UC. Notably, the hormone is a potent antioxidant that is naturally synthesized by mammals. It has the ability to limit OS in lipid and aqueous cellular environments, which occurs as a consequence of crossing physiological barriers (e.g., the mitochondrial membrane). Notably, melatonin is responsible for scavenging peroxyl and hydroxyl radicals, and it performs a protective function in the preliminary and late stages of numerous adverse health conditions that involve ROS metabolites.

### 2.7. Extracellular Antioxidants

Certain antioxidant enzymes are reliant on minerals including Zn, Fe, Cu, and Mn. Variable SOD isoforms need Zu, Cu, or Mn to serve as cofactors, and iron must be present for effective CAT activity to occur [14]. Plants are the major source of polyphenols, extracellular antioxidants that are primarily found in fruits and vegetables [53].

Vitamins A, C, and E are antioxidants, and deficiencies of each are frequently identified in patients with UC and CD. This is primarily attributed to malnutrition from an absence of fruits and vegetables [54]. Yellow fruits and green vegetables contain vitamin A or *β*-carotene (pro-vitamin A), which is noteworthy for its antioxidant activity, which relies on retinol-binding proteins. Vitamin C is synthesized from glucose, has the ability to reduce numerous types of RO, and can be derived from fresh fruits and vegetables. Vitamin E plays a protective role for the cell membrane with respect to lipid peroxidation.

## 3. Conclusions

Because OS is presently considered as a potentially critical pathogenic factor in the pathogenesis, progression, and severity of IBD, its prior status as the result of chronic inflammation of the intestinal mucosa in facilitating pathological change within the GI tract is understudied in IBD patients. Even for adults, the foundational mechanisms that give rise to the condition and underpin its likely course of development must still be clarified. Clinical research into currently used and experimental anti-IBD therapies, particularly those that include naturally occurring antioxidants, is associated with positive patient outcomes for both IBD and colorectal cancer [55]. Nevertheless, given that a range of intestinal variables (such as bacteria, digestive enzymes, and food metabolites) can have an effect on the antioxidative characteristics of drugs (often rendering them ineffective), additional clinical trials are required. It is anticipated that a renewed understanding of the fundamental antioxidative impacts of existing pharmaceuticals and proposed experimental agents will aid in the provision of increasingly more satisfying and effective healthcare services to new and relapsed IBD patients. Furthermore, with the derivation of novel antioxidant-heightening interventions, paired with the administration of traditional pharmaceuticals, it is expected that patients with IBD will benefit from significantly more favorable outcomes promoted by further clinical trials.
